# Microbial community changes mediate the decline in soil organic carbon mineralization with depth in tea plantations

**DOI:** 10.1186/s13021-026-00485-7

**Published:** 2026-07-13

**Authors:** Shaobo Zhang, Yifeng Xie, Hongbing Zhang, Junyan Lv, Weizhen Wu, Wenyan Han, Gaodi Zhu, Qiuhong Wang, Claudien Habimana Simbi, Rongxiu Yin, Xin Li

**Affiliations:** 1 Tea Research Institute, Chinese Academy of Agricultural Sciences, Hangzhou, 310000 China; 2 State Key Laboratory of Subtropical Silviculture, Zhejiang A & F University, Hangzhou, 311300 China; 3 State Key Laboratory of Tea Plant Germplasm Innovation and Resource Utilization, Hangzhou, 310000 China; 4 Qingyuan County Agriculture and Rural Bureau, LiShui, 323000 China; 5 Agricultural Information Institute, Chinese Academy of Agricultural Sciences, Beijing, 100081 China; 6 Tea Research Institute, Guizhou Provincial Academy of Agricultural Sciences, Guiyang, 550006 China

**Keywords:** Tea plantations, Soil organic carbon sequestration, Cellulose-degrading Carbon-cycling functional genes, Microbial community

## Abstract

**Background:**

Tea plantation soils serve as vital carbon (C) sinks rich in soil organic carbon (SOC), yet existing research primarily focuses on the 0–20 cm topsoil, leaving a lack of systematic study of SOC mineralization characteristics and microbial regulatory mechanisms across the full 0–100 cm soil profile. Therefore, we conducted incubation experiments with typical tea plantation soils to clarify changes in SOC mineralization, the SOC pool, the microbial community, and the functional genes (such as *GH48* and *cbh*I) and enzyme activities related to C decomposition at five soil depths (0–20 cm, 20–40 cm, 40–60 cm, 60–80 cm, and 80–100 cm), and to elucidate their relationships, thereby revealing the mechanisms affecting SOC mineralization in different soil layers.

**Results:**

The results revealed significant declines in SOC mineralization rate (from 842 to 431 mg kg⁻¹), particulate organic carbon, water-soluble organic carbon, microbial biomass carbon, β-glucosidase (from 71.6 to 18.2 µg g^− 1^ h^− 1^ at the end of incubation), cellobiohydrolase (from 0.229 to 0.091 mg g^− 1^ 3d^− 1^ at the end of incubation), *GH48* and *cbh*I gene abundances (decreased from 9.2 × 10^7^ to 1.8 × 10^7^ and 7.6 × 10^7^ to 1.4 × 10^6^ copies g^− 1^ at the end of incubation, respectively), respectively with increasing soil depth. The decrease in the SOC mineralization rate with increasing soil depth was significantly associated with declines in the labile C fraction, C-decomposition-related extracellular enzyme activity, and functional gene abundance. Additionally, increasing soil depth significantly altered the microbial community structure and composition, particularly the relative abundances of dominant taxa such as Alphaproteobacteria, Bacilli, Sordariomycetes, Tremellomycetes, Mortierellomycetes, and Dothideomycetes, thereby further influencing SOC mineralization rate.

**Conclusions:**

Our findings demonstrate that increasing soil depth significantly alters soil microbial community characteristics, particularly by reducing the abundance of C-degrading functional genes and enzyme activities, thereby lowering SOC mineralization rate and attenuating soil carbon emissions. These results underscore the importance of deep tillage during fertilization and the incorporation of pruning residue to enhance SOC sequestration in tea plantations.

**Supplementary Information:**

The online version contains supplementary material available at https://doi.org/10.1186/s13021-026-00485-7.

## Introduction

Soil organic carbon (SOC) pools are essential components of terrestrial ecosystems, capable of storing carbon (C) at levels two to three times those of the combined atmospheric and vegetation C reservoirs [17, 25, 86]. Accordingly, its dynamic changes directly affect the global C balance and climate change [12, 33, 34, 53, 65]. As one of the critical agricultural ecosystems, SOC storage is not only related to tea yield and quality but also serves as the key carrier of the carbon sink function of the tea plantation ecosystem [74, 89]. Research indicates that SOC storage in Chinese tea plantations reaches 207.13 Tg in the 0–20 cm layer. The SOC storage of the soil layer reached 124.58 Tg, and the average SOC density was about 59.17 Mg ha^–1^ [74]. However, intensive management practices characteristic of tea cultivation—such as long-term, high-dose organic and inorganic fertilizer inputs, soil acidification, pruning, etc., will significantly alter the turnover rate of SOC in tea plantations, thereby affecting its C sink potential [85, 89]. Most studies investigating SOC pool dynamics have focused on the top 20 cm of soil, likely because this layer is directly influenced by human activities and soil management practices [4, 45, 91, 97]. Nevertheless, worldwide data show that SOC stocks are 863 and 1824 billion tonnes, respectively, in the 1–30 cm and 0–100 cm soil profiles [56]. Specifically in tea plantation ecosystems, the bulk of domestic and overseas research focuses on topsoil, and comparative studies of SOC turnover across the full 0–100 cm soil profile are largely absent. Therefore, elucidating the characteristics and mechanisms of SOC transformation across different soil layers is crucial for enhancing the capacity of soil to sequester SOC in tea plantation ecosystems.

SOC mineralization is an essential microbially driven process that influences soil C reserves, which are closely related to changes in the vertical stratification of SOC components and microbial communities in tea plantation soils [46, 57, 63, 87, 89]. Specifically, easily decomposable organic C components such as particulate organic carbon (POC) in soils usually drive rapid mineralization of SOC, whereas an increase in the content of stable organic C components such as mineral-associated organic carbon (MAOC) is beneficial for slowing down the rate of SOC mineralization [22, 31, 32, 35, 61, 96]. As a typical perennial artificial ecosystem, the tea plantation ecosystem has a high and stable exogenous C input pathway, such as the return of pruning material to the field and the application of organic fertilizer, which makes its surface SOC storage advantage prominent [89]. However, most tea gardens use no-tillage or surface tillage in their management practices, and these measures will significantly alter the composition and content of organic C across different soil layers of tea plantations [26, 30, 48, 81, 82]. Thus, a comprehensive analysis of how SOC fractions vary with soil depth and their impact on mineralization is essential for advancing our understanding of SOC stabilization and improving strategies to enhance SOC storage in tea plantation soil.

Crucially, management measures, such as the continuous input of exogenous organic matter, will also significantly affect the structure and composition of microbial communities across different soil layers in plantation gardens [5, 67, 85]. Changes in microbial community structure and functional microbial composition will significantly affect the mineralization intensity of SOC [13, 46, 58, 73]. For example, changes in the relative abundance of specific taxa (e.g., *Umbelopsidomycetes*) and in the expression of C-cycle functional genes (e.g., *GH48* and *cbh*I, encoding cellulase) directly regulate the rate of SOC decomposition [38, 43, 59, 91]. In the unique tea plantation habitat, characterized by continuous organic matter input and pronounced soil acidification, the long-term management practice combining shallow tillage with surface mulching of tea pruning residues results in a heterogeneous vertical distribution of SOC across soil profiles. Nevertheless, the intrinsic correlations among SOC fractions across soil layers, microbial community composition, and C-degrading functional genes, as well as the profile differentiation mechanism by which their coupled interactions regulate SOC mineralization rates, remain poorly understood.

Tea (*Camellia sinensis* L.) is a valuable economic crop, and the area under tea cultivation in China exceeded 3.43 million hectares by 2024 [6]. Previous investigations demonstrated that tea plantation soil depth profiles exhibit significant differences in SOC content and in the structure and composition of the microbial community [67, 85]. However, the characteristics of the SOC mineralization rate across different soil layers of a tea plantation, particularly the interconnections among SOC components, the microbial community, and mineralization rate, remain insufficiently investigated.

Therefore, this study collected tea plantation soil samples across multiple depth intervals and conducted incubation experiments to investigate two research goals: (1)

explore the mineralization characteristics of SOC across different soil layers of the tea plantation, and (2) reveal the biotic and abiotic mechanisms that affect the mineralization of SOC across different soil layers. Specifically, we hypothesize:

1. With increasing soil depth, the content of easily decomposable SOC and the mineralization rate of SOC in soil decrease;
2. The decline in the SOC mineralization rate in deep soil layers is mainly due to changes in the structure of microbial communities, particularly a reduction in the number of functional genes and enzyme activities related to C decomposition.

This study systematically explores the dynamics of SOC mineralization and the C sequestration potential of the 0–100 cm soil profile, overcoming the limitation of existing tea garden studies that are restricted to topsoil. It elucidates the differential mechanisms of SOC turnover in topsoil and subsoil by integrating C components, C-decomposing enzymes and functional genes, along with high-throughput microbial sequencing, which lays a theoretical foundation for the research and development of C sequestration strategies in tea gardens, as well as for subsoil C management.

## Materials and methods

### Site description and soil sampling

Soil samples of the incubation study were collected from a tea farm across five depth intervals (0–20, 20–40, 40–60, 60–80, and 80–100 cm) in Meijiawu Village (30°10’N, 120°5’E), Xihu District, Hangzhou City, China. The research site is 153 m above mean sea level. The area experiences a humid subtropical climate with monsoonal influence, with an average yearly temperature of 17.0 °C, and 1533 mm of precipitation annually. The soil is classified as an Ultisol, formed from loamy clay derived from Anshan quartz-free porphyry formations [24]. The soil pH values of the five soil layers were 3.86, 3.89, 3.92, 4.08 and 4.14, respectively. Their total carbon contents were 3.69%, 1.78%, 1.07%, 0.704% and 0.85%, while the total nitrogen contents were 0.39%, 0.21%, 0.18%, 0.11% and 0.073%.

The tea cultivation system, established more than a century ago, grows the Longjing (*C. sinensis* L.) variety, with an annual spring tea production of 175 kg ha^− 1^ a^–1^. Following spring tea harvesting, tea shrubs undergo uniform pruning, and the resulting pruning materials are subsequently incorporated back into the cultivation area. The nitrogen, phosphorus, and potassium ratio in tea plantation fertilization is 3:1:1, with annual nitrogen fertilizer application in the tea cultivation system reaching approximately 460 kg N ha^− 1^. Organic fertilization is implemented at roughly 12,000 kg ha⁻¹ yearly, consisting primarily of farmyard manure and rapeseed cake materials. SOC concentrations demonstrated a gradual reduction with increasing depth, recording 3.69%, 1.22%, 0.86%, 0.31%, and 0.31% at 0–20 cm, 20–40 cm, 40–60 cm, 60–80 cm, and 80–100 cm soil horizons, respectively.

### Incubation experiment

The incubation trial encompassed five treatments according to soil horizon depth: (1) 0–20 cm, (2) 20–40 cm, (3) 40–60 cm, (4) 60–80 cm, and (5) 80–100 cm. Each treatment was performed in triplicate.

The experimental soil was collected by digging a profile in the selected tea plantation, and the fresh soil was brought back to the laboratory for root-picking treatment. Prepare fresh soil to a mass of 400 g oven-dried per replicate. Each soil specimen was divided into two segments: 200 g of soil designated for CO_2_ quantification and an additional 200 g allocated for destructive sampling protocols. The soil was positioned in 500 mL glass vessels at a mass density of 1.3 g cm⁻³, maintaining a water-holding capacity of 60%.

CO₂ efflux was quantified employing the alkali absorption–titration methodology. Specifically, a soil vessel and a small container holding 10 mL of 0.2 mol L^− 1^ NaOH were placed in 500 mL sealed bottles. The soils were incubated at 25 °C for 45 days. To maintain consistent moisture conditions, water was added to each CO₂ quantification using a gravimetric approach to ensure a 60% WHC. Subsequently, three blank 500 mL containers were prepared using the same methodology as the experimental containers to account for headspace CO_2_ within sealed bottles. Following NaOH trap replacement, CO_2_ quantifications were conducted on days 1, 3, 5, 10, 15, 30, and 45. Titration against HCl (0.2 mol L^− 1^) solution was employed to determine how much CO₂ the NaOH solution absorbed. Destructive sampling protocols were implemented on days 15 and 45 to determine the composition of the SOC pool, chemical architecture, enzymatic activity, and microbial community characteristics.

### Soil physicochemical properties

WSOC and MBC measurements followed methodologies established by Zhou et al. [95]. POC and MAOC determinations utilized fractionation approaches described by Cambaradella and Elliott [8]. Enzymatic functions in soil, including β-glucosidase and cellobiohydrolase assays, were performed following protocols outlined in Alef [1] and Zhang et al. [92], respectively. Detailed methodological procedures are documented in the appendix.

### Microbial community and C-cycling genes analysis

DNA extraction was conducted on triplicate 0.2 g soil specimens utilizing Fast DNA SPIN isolation kits (MP Biomedicals, USA), subsequently processed through OMEGA purification protocols (Omega Bio-Tek, USA). Genetic material concentration and quality assessment were executed using NanoDrop ND-1000 spectrophotometric analysis (Thermo Fisher, Waltham, MA, USA). Extracted genetic material was stored at -70 °C pending subsequent molecular analysis. Amplification of fungal internal transcribed spacer (ITS) sequences was achieved through ITS3F/ITS4R primer configurations [50], whereas bacterial 16 S ribosomal RNA (rRNA) genetic regions were amplified via 515 F/806R primer systems [20]. Polymerase chain reaction procedures were executed using an ABI GeneAmp^®^ 9700 thermal cycling apparatus (Applied Biosystems, USA) within 20 µL reaction volumes incorporating TransStartFastpfu DNA polymerase. The thermal protocol initiated with 95 °C denaturation lasting 180 s, followed by 35 amplification rounds (ITS) or 27 rounds (16 S rRNA) comprising 95 °C denaturation (30s), 55 °C primer binding (30s), and 72 °C elongation (45s), concluding with 72 °C final elongation for 600s.

Amplified products processed for next-generation sequencing using purified libraries. Amplified products processed for high-throughput sequencing using purified libraries constructed with TruSeqTM DNA library construction methods. Paired-end amplicon sequencing was conducted on Nextseq 2000 platforms with FASTQ format data archiving, followed by sequence refinement. Bioinformatics analysis employed QIIME2 software, incorporating the DADA2 algorithm, for quality assessment and feature table construction [7]. Sequences were organized into amplicon sequence variants (ASVs) and taxonomically classified using Silva 138 databases [52]. Raw datasets are available through Sequence Read Archive (SRA) under accessions PRJNA1085977 and PRJNA1085896. C-cycling functional gene quantification was conducted to expand analytical coverage; comprehensive methodologies are documented in Supplemental Information.

### Statistical analysis

Treatment comparisons utilized one-way analysis of variance (ANOVA) accompanied by least significant difference (LSD) post-hoc evaluations. Initial assessments confirmed variance homogeneity and normality requirements, with logarithmic transformations implemented when needed to correct heteroscedasticity. Spearman correlation coefficients were calculated to establish relationships between cumulative CO_2_ releases, microbial indicator taxa, and soil characteristics. Statistical computations were employed SPSS version 18.0 (IBM, Inc., USA).

Microbial community analyses were conducted using R version 4.1.2 (R Core Team, 2021). Alpha diversity calculations employed Phyloseq packages for Shannon indices, Chao1 estimators, and ASV counts [47]. ASV matrices underwent Hellinger transformations, with community structural differences assessed via the Bray-Curtis dissimilarity metric was used to construct the distance matrix for principal coordinate analysis (PCoA). Relationships between environmental factors and bacterial and fungal communities were examined using Mantel tests based on Bray-Curtis distance matrices for microbial community data and Euclidean distance matrices for environmental variables, with significance assessed via 999 permutations.

## Results

### SOC mineralization

**Fig. 1:**
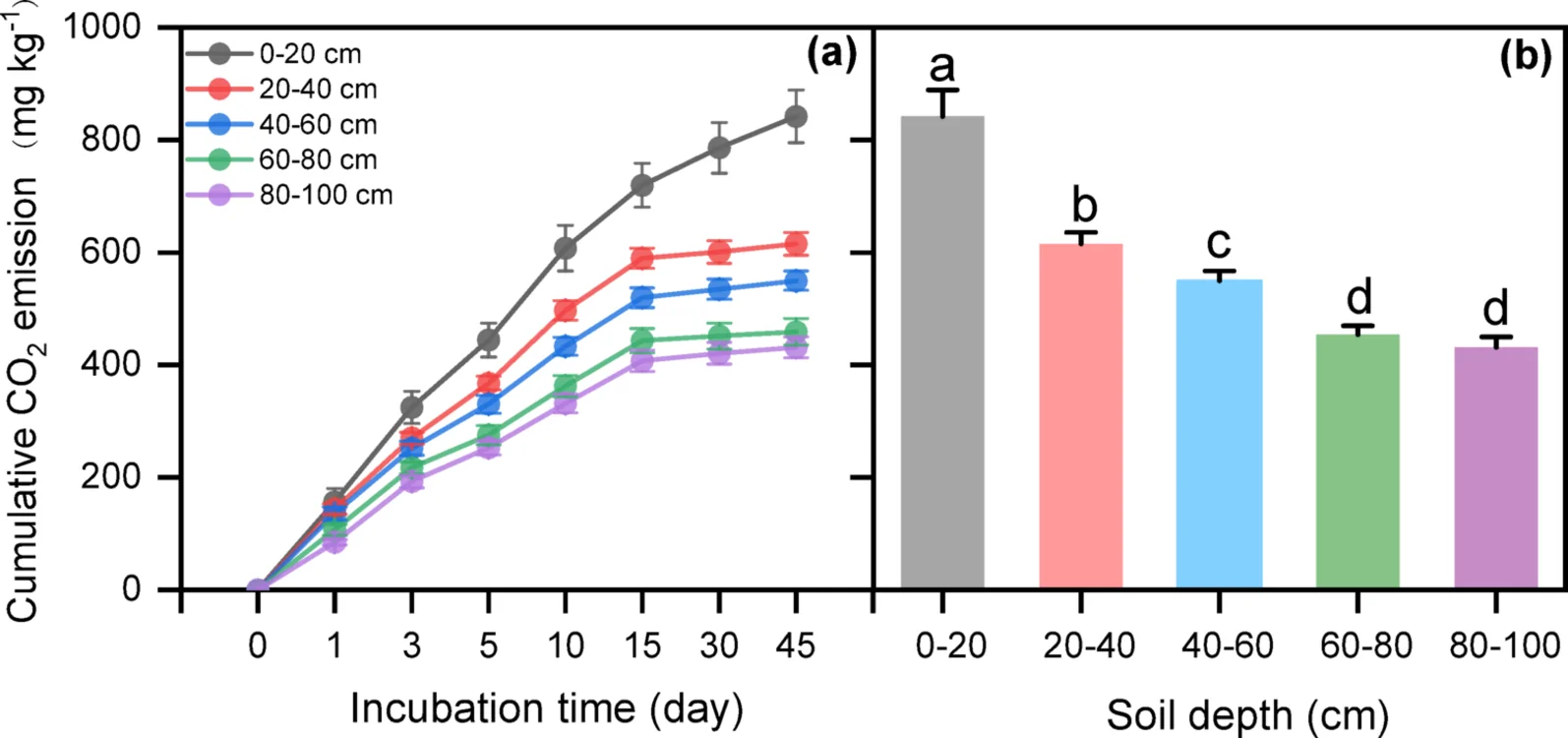
Effects of soil depths on the dynamics of the cumulative CO_2_ emission from tea plantation soil during the 45-d incubation period. **a** Dynamics of cumulative CO_2_ emission per kg of soil, **b** 45-d cumulative CO_2_ emission per kg of soil (*P* < 0.05). 0–20, 20–40, 40–60, 60–80, and 80–100 represent the soils from five distinct soil layers. Error bars indicate standard deviations. Different letters above the bars indicate significant differences between treatments (*P* < 0.05)

As soil horizon depth increased throughout the tea plantation ecosystem, cumulative SOC mineralization rate showed pronounced (*P* < 0.05) reductions (Fig. 1a). Total CO₂ efflux from substrate layers spanning 0–20 cm, 20–40 cm, 40–60 cm, 60–80 cm, and 80–100 cm horizons measured 842, 615, 550, 459, and 431 mg kg⁻¹, correspondingly (Fig. 1b). Additionally, aggregate CO₂ release patterns revealed a statistically significant hierarchy (*P* < 0.05) following the sequence: 80–100 cm = 60–80 cm < 40–60 cm < 20–40 cm < 0–20 cm depth intervals (Fig. 1b).

### The pool of SOC

**Fig. 2:**
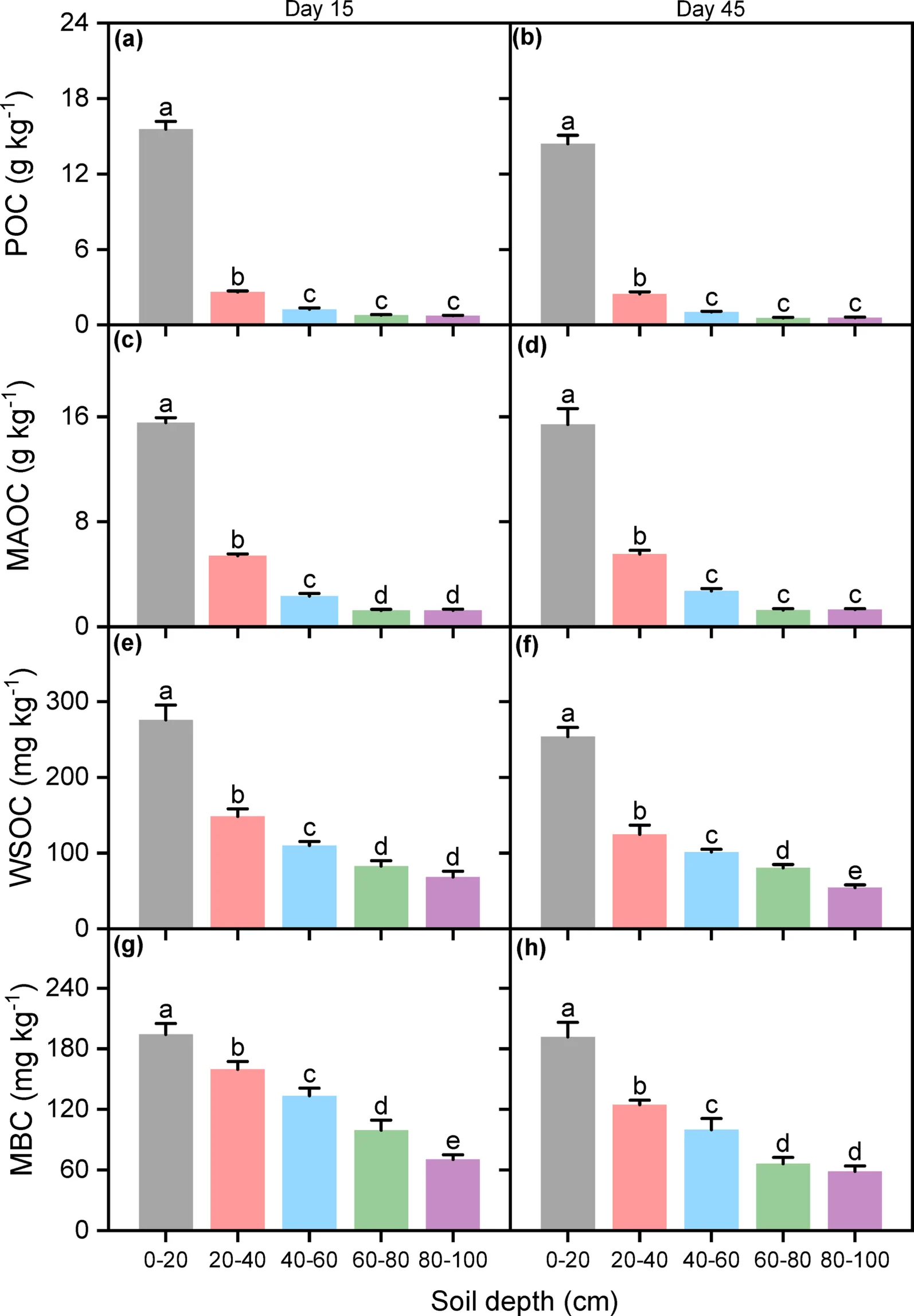
Effects of soil depths on soil particulate organic carbon (POC) (**a**-**b**), mineral-associated organic carbon (MAOC) (**c**-**d**), water-soluble organic C (WSOC) (**e**-**f**), and microbial biomass C (MBC) (**g**-**h**) in tea plantation soil under the 15 and 45 incubation time. 0–20, 20–40, 40–60, 60–80, and 80–100 represent the soils from five distinct soil layers. Different letters indicate significant differences between treatments (*P* < 0.05)

Throughout all incubation periods, surface-layer (0–20 cm) POC concentrations remained consistently elevated (*P* < 0.05) relative to deeper zones, while intermediate depths (20–40 cm) showed significantly higher (*P* < 0.05) POC levels than the three deepest layers (40–60, 60–80, 80–100 cm) (Fig. 2a, b). At day 15, MAOC concentrations followed this pattern (*P* < 0.05): 0–20 cm > 20–40 cm > 40–60 cm > 60–80 cm = 80–100 cm (Fig. 2c). By day 45, this hierarchy shifted (*P* < 0.05) to: 0–20 cm > 20–40 cm = 40–60 cm = 60–80 cm = 80–100 cm (Fig. 2d).

At the 15-day timepoint, water-soluble organic carbon levels demonstrated the hierarchical arrangement (*P* < 0.05): 0–20 cm > 20–40 cm > 40–60 cm > 60–80 cm = 80–100 cm (Fig. 2e). By reaching day 45, this distribution pattern shifted (*P* < 0.05) to establish: 0–20 cm > 20–40 cm > 40–60 cm > 60–80 cm > 80–100 cm (Fig. 2f). MBC levels during the 15-day period manifested the progression (*P* < 0.05): 0–20 cm > 20–40 cm > 40–60 cm > 60–80 cm > 80–100 cm (Fig. 2g). This sequential arrangement continued through day 45 (Fig. 2h).

### Carbon cycle-related enzyme activity in soil

**Fig. 3:**
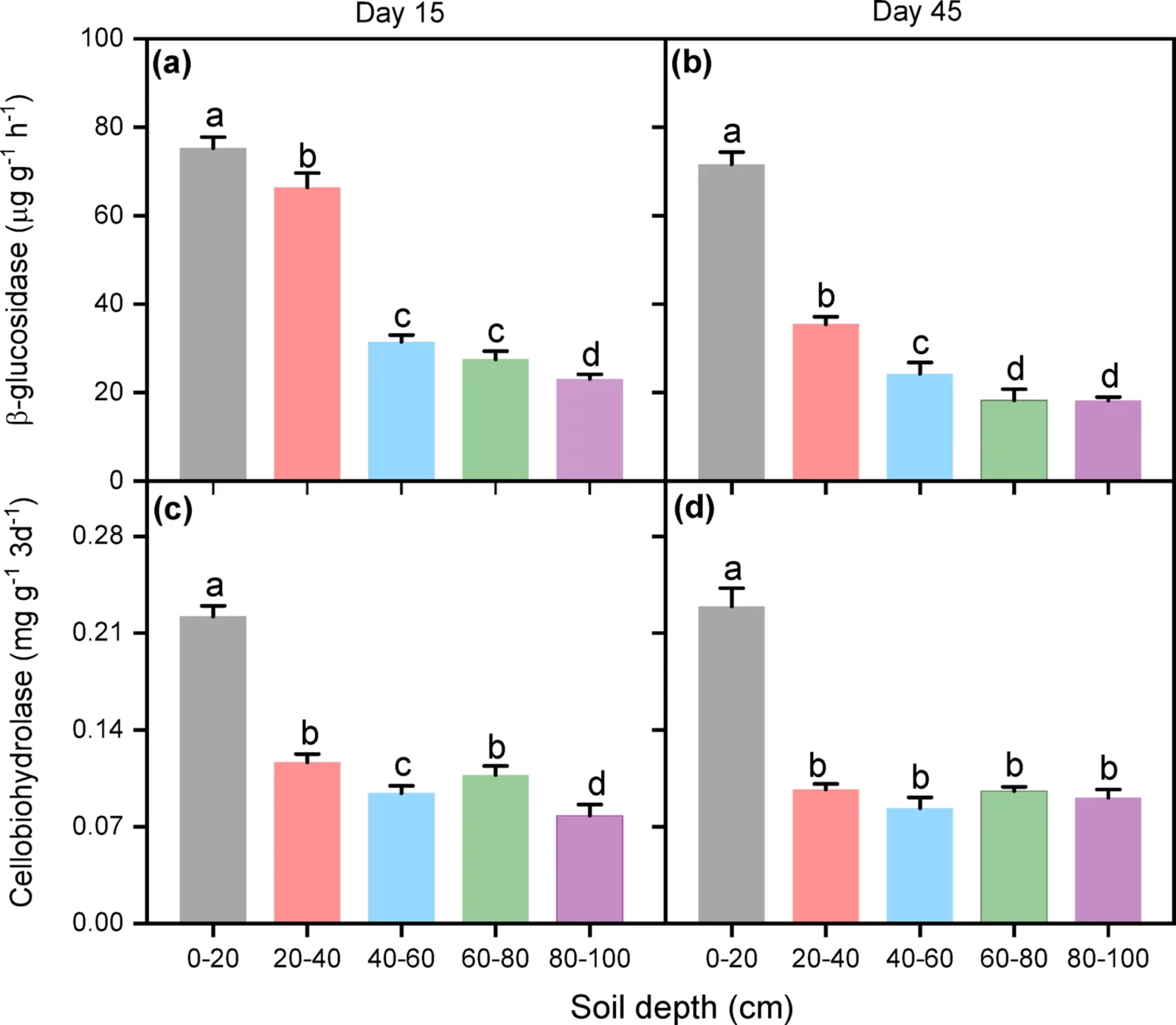
Effects of soil depths on soil **a**, **b** β-glucosidase and **c**, **d** cellobiohydrolase activity in tea plantation soil. Error bars denote standard deviations. 0–20, 20–40, 40–60, 60–80, and 80–100 represent the soils from five distinct soil layers. Different letters above the bars indicate significant differences between treatments (*P* < 0.05)

Compared to surface soils (0–20 cm), β-glucosidase activity in subsurface layers (20–40, 40–60, 60–80, 80–100 cm) declined significantly (*P* < 0.05) by 12%, 58%, 63%, and 69%, respectively, at day 15. Statistical differences occurred among all treatments except between the 40–60 and 60–80 cm depths (Fig. 3a). By day 45, β-glucosidase activity in these same layers decreased significantly (*P* < 0.05) by 50%, 66%, 75%, and 75%, respectively, relative to surface soils. All treatments differed significantly except for the two deepest layers (Fig. 3b).

At day 15, cellobiohydrolase activity showed significant (*P* < 0.05) differences among all depths except between the 20–40 and 60–80 cm layers, displaying declining trends with increasing depth (Fig. 3c). By day 45, surface layer cellobiohydrolase activity remained significantly elevated (*P* < 0.05) compared to all deeper layers (Fig. 3d).

### Carbon cycle-related functional gene abundance in soil

**Fig. 4:**
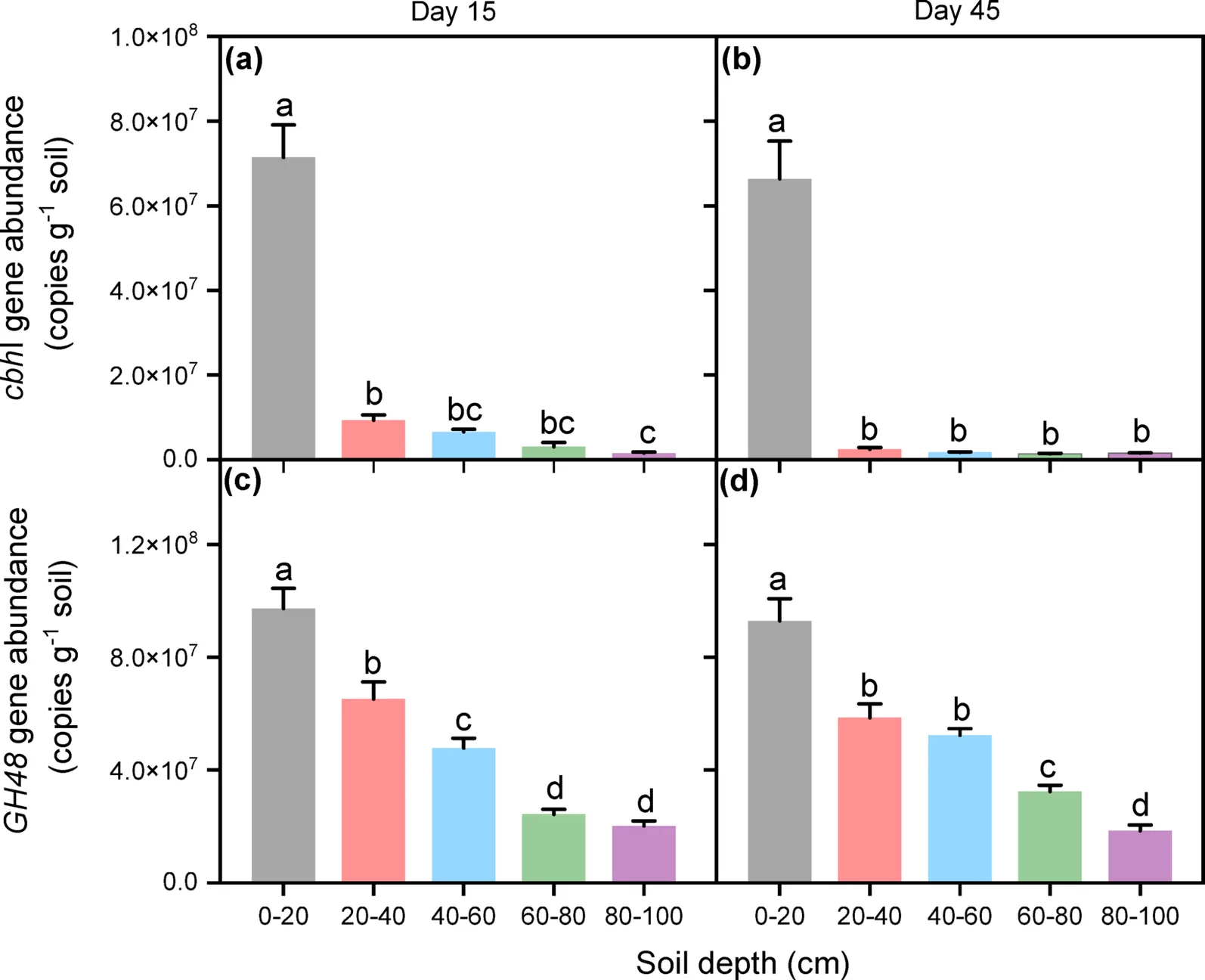
Effects of soil depths on soil **a**, **b** *cbh*I and **c**, **d** *GH48* gene abundance in a tea plantation. Error bars represent standard deviations. 0–20, 20–40, 40–60, 60–80, and 80–100 represent the soils from five distinct soil layers. Different letters above the bars indicate significant differences between treatments (*P* < 0.05)

Throughout both the 15- and 45-day incubation intervals, gene copy quantities for *cbh*I and *GH48* were significantly lower across all soil horizons beneath the 0–20 cm horizon (Fig. 4a-d). At the 15-day timepoint, *cbh*I gene abundance within the 80–100 cm soil horizon was significantly lower than in the 20–40 cm, 40–60 cm, and 60–80 cm horizons (Fig. 4a). Concerning *GH48* gene abundance, its distribution at the 15-day timepoint displayed the sequence: 20–40 cm > 40–60 cm > 60–80 cm = 80–100 cm. At the 45-day timepoint, this arrangement was modified slightly to: 20–40 cm = 40–60 cm > 60–80 cm > 80–100 cm (Fig. 4c-d).

### Soil microbial community composition

Bacterial diversity (Shannon index) in the deepest layer (80–100 cm) was significantly lower (*P* < 0.05) than in the 20–40 and 40–60 cm layers on day 15, whereas the 40–60 cm depth showed significantly higher (*P* < 0.05) diversity than surface layers on day 45 (Table 1).

Fungal diversity (Shannon index) in the 60–80 cm layers significantly exceeded (*P* < 0.05) that of the surface, 20–40, and 80–100 cm layers on day 15, with the surface layer showing the lowest values across depths. By day 45, surface fungal diversity was significantly lower (*P* < 0.05) than at all other depths, except at 20–40 cm. Fungal richness (Chao index) was significantly higher (*P* < 0.05) in the 20–40 and 40–60 cm layers than in the other depths at day 15, whereas only the 40–60 cm layer showed significantly higher (*P* < 0.05) richness than the other depths at day 45 (Table 1).

**Table 1:** Effects of soil depths on microbial diversity after 15 and 45 d of incubation in the tea plantation soil microbiome

	Bacterial Shannon index			Bacterial Chao1 index	
	Day 15	Day 45		Day 15	Day 45
0–20 cm^†^	2.48 ± 0.06ab^‡^	2.50 ± 0.10b		58.7 ± 2.1a	58.1 ± 0.1a
20–40 cm	2.63 ± 0.02a	2.61 ± 0.04ab		62.6 ± 3.3a	59.9 ± 2.4a
40–60 cm	2.65 ± 0.02a	2.68 ± 0.04a		65.5 ± 2.2a	64.7 ± 4.7a
60–80 cm	2.57 ± 0.06ab	2.63 ± 0.01ab		62.4 ± 2.1a	64.3 ± 5.3a
80–100 cm	2.41 ± 0.11b	2.58 ± 0.04ab		63.1 ± 4.6a	66.7 ± 2.0a

	Fungal Shannon index			Fungal Chao1 index	
	Day 15	Day 45		Day 15	Day 45
0–20 cm	^†^1.69 ± 0.04c	1.69 ± 0.06b		32.0 ± 1.0b	29.0 ± 1.0b
20–40 cm	2.00 ± 0.01b	1.92 ± 0.03ab		36.9 ± 0.8a	29.8 ± 0.3b
40–60 cm	2.13 ± 0.03ab	2.11 ± 0.06a		36.5 ± 0.9a	34.2 ± 0.8a
60–80 cm	2.19 ± 0.05a	2.12 ± 0.07a		30.0 ± 0.9b	28.3 ± 1.5b
80–100 cm	2.00 ± 0.11b	1.95 ± 0.16a		30.3 ± 1.5b	29.0 ± 1.7b

The mean values after “±” represent the standard deviation

†0–20 cm, 20–40 cm, 40–60 cm, 60–80 cm, 80–100 cm represent five distinct soil layers

‡ Different lowercase letters indicate significant differences between treatments (*P* < 0.05)

**Fig. 5:**
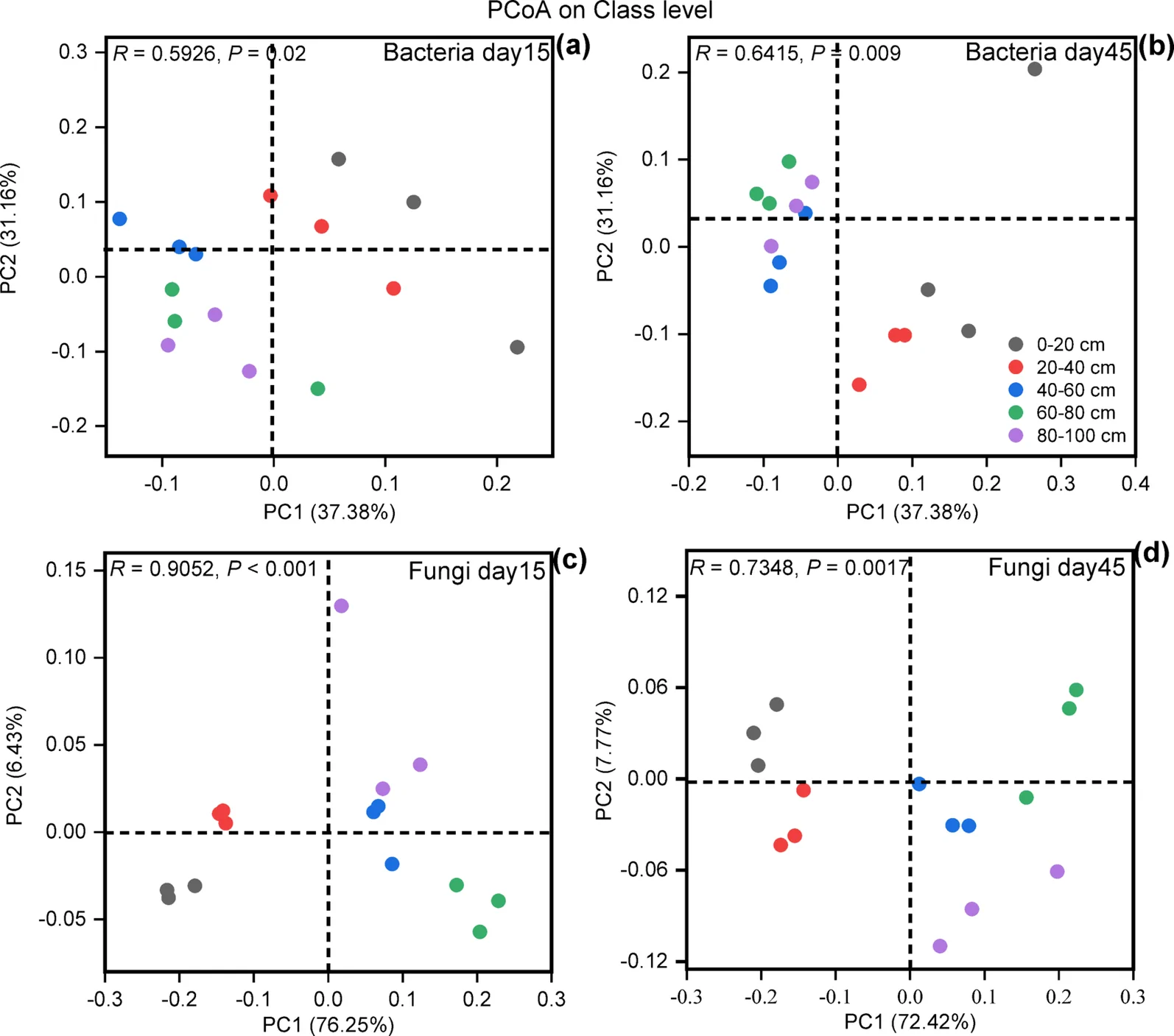
PCoA representing variations between bacterial **a**, **b** and fungal **c**, **d** community compositions in tea plantation soil under the different soil depth Bray–Curtis distance. 0–20, 20–40, 40–60, 60–80, and 80–100 cm represent the soils from five distinct soil layers

Principal coordinate analysis (PCoA) showed significant differences in bacterial communities across all depths at day 15, except in the 60–80 and 80–100 cm layers. At day 45, significant differences were observed between the surface and 20–40 cm layers, with both showing substantial differences from all subsurface layers (Fig. 5a, b). Fungal communities also showed significant differences across all depths at both time points.

**Fig. 6:**
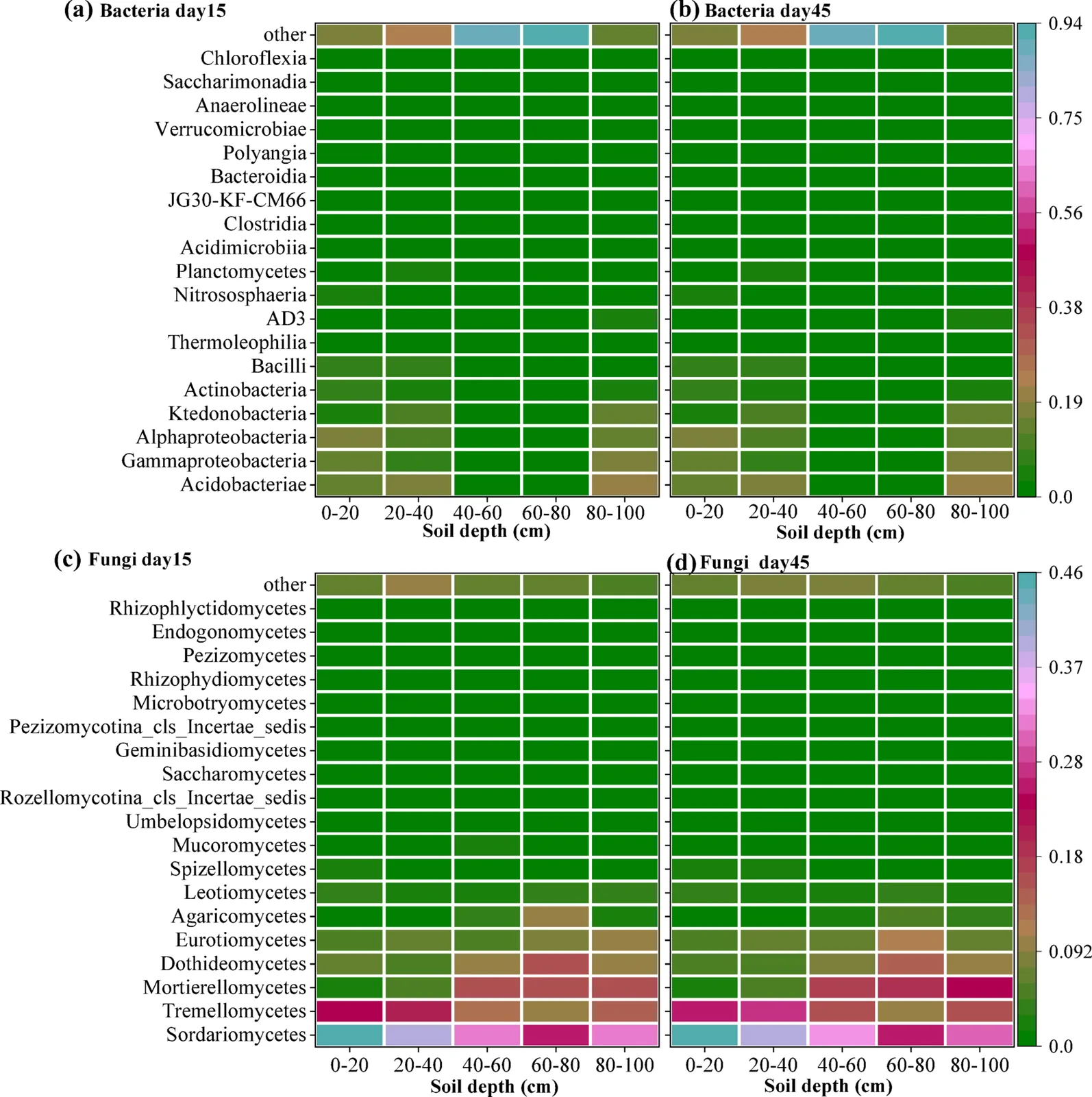
Compositions of bacterial **a**, **b** and fungal **c**, **d** communities in a tea plantation soil at the different soil depths. The names of the classes with a > 1% relative abundance are shown. 0–20, 20–40, 40–60, 60–80, and 80–100 represent the soils from five distinct soil layers

Bacterial Alphaproteobacteria and Bacilli relative abundances decreased significantly with depth progression at both timepoints. Conversely, Ktedonobacteria and AD3 relative abundances increased with depth (Fig. 6a, b). Relative abundances of the fungal orders Sordariomycetes and Tremellomycetes declined significantly with increasing depth. In contrast, Mortierellomycetes and Dothideomycetes showed significantly increasing abundances along depth gradients at day 15, and this trend continued through day 45 (Fig. 6c, d).

### Relationships between soil properties, microbial community compositions, and soil CO_2_ emissions

**Fig. 7:**
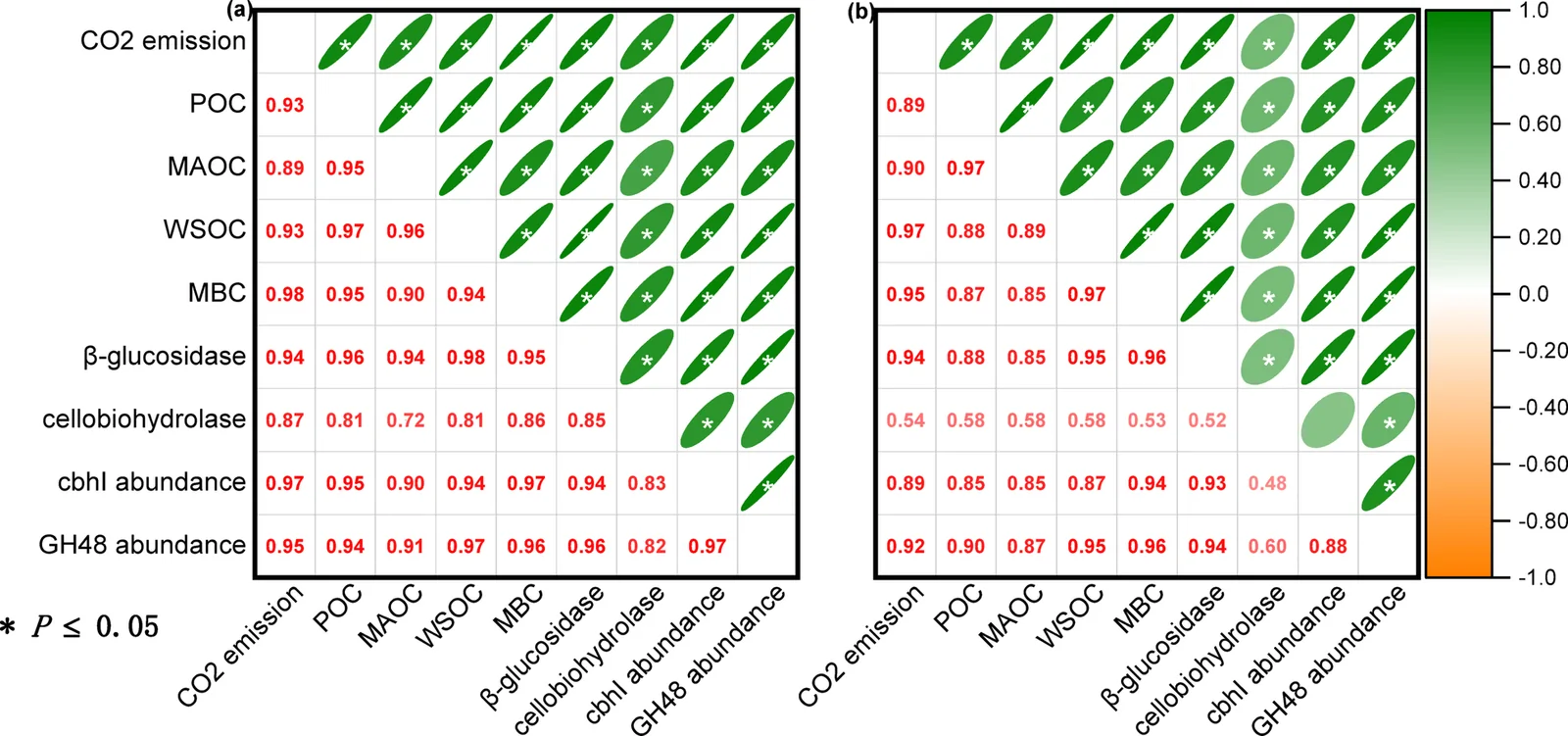
Relationships between soil organic carbon mineralization rate and environmental factors, including the contents of particulate organic carbon (POC), mineral-associated organic carbon (MAOC), water-soluble organic C (WSOC), microbial biomass C (MBC), β-glucosidase activity, cellobiohydrolase activity, *cbh*I gene abundance, and *GH48* gene abundance in tea plantation soil under different soil depths on day 15 (a) and day 45 (b)

**Fig. 8:**
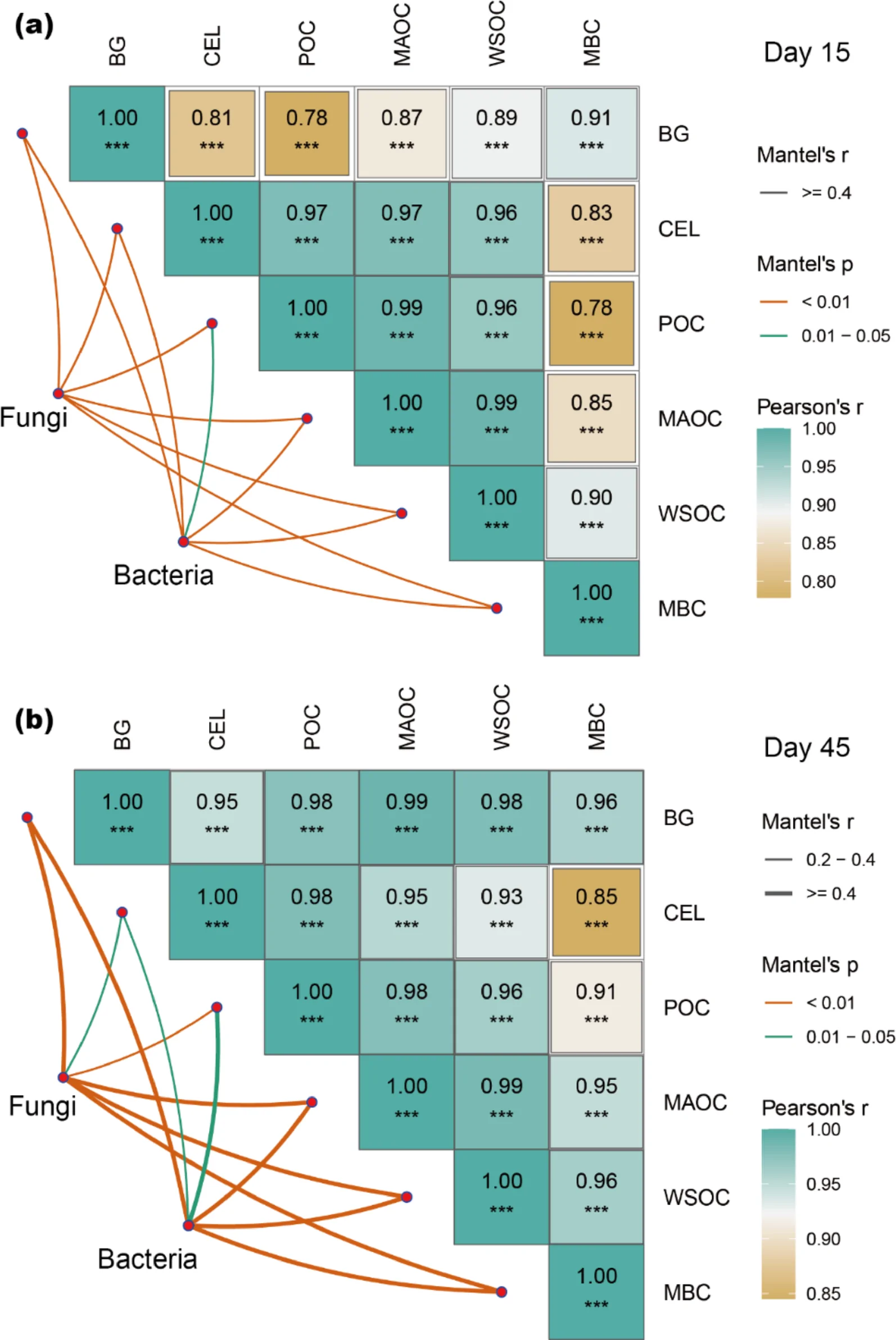
Mantel test representing variations in soil environmental factors and the community compositions of bacteria and fungi in tea plantation soil under different soil depths on day 15 (a) and day 45 (b). Graduated colors illustrate Pearson’s correlation coefficient, the thickness of connecting lines matches Mantel’s r statistic, and color tones indicate statistical significance.*** Extremely significant at the 0.001 significance level

Independent of incubation duration, cumulative soil CO_2_ efflux demonstrated positive (*P* < 0.01) associations with POC, MAOC, WSOC, MBC concentrations, β-glucosidase and cellobiohydrolase (*P* < 0.01; *P* < 0.05) enzymatic activities, along with *cbh*I and *GH48* gene abundances (Fig. 7a and b). Additionally, cumulative soil CO_2_ efflux exhibited positive relationships with Alphaproteobacteria, Bacilli, Clostridia, Sordariomycetes, and Tremellomycetes abundances, while displaying negative associations with bacterial Chao1 diversity index, fungal Shannon diversity index, and proportional abundances of Ktedonobacteria, AD3, JG30-KF-CM66, Bacteroidia, Mortierellomycetes, Dothideomycetes, Eurotiomycetes, and Leotiomycetes (*P* < 0.05; Table 2). Moreover, Mantel test results revealed that fluctuations in soil POC, MAOC, WSOC, and MBC concentrations significantly shaped both bacterial and fungal community architectures at the 15- and 45-day time points (Fig. 8).

**Table 2:** Correlation coefficients between the relative abundance of soil bacterial and fungal classes and cumulative CO_2_ emissions in a tea plantation soil (Spearman’s rank correlation coefficient)

Bacterial	Cumulative CO_2_ emission	Fungal	Cumulative CO_2_ emission
Chao1 index	-0.536**	Chao1 index	0.196
Shannon index	-0.056	Shannon index	-0.600**
Acidobacteriae	-0.252	Sordariomycetes	0.777**
Gammaproteobacteria	-0.503**	Tremellomycetes	0.772**
Alphaproteobacteria	0.438*	Mortierellomycetes	-0.768**
Ktedonobacteria	-0.512**	Dothideomycetes	-0.711**
Actinobacteria	-0.353	Eurotiomycetes	-0.569**
Bacilli	0.798**	Agaricomycetes	-0.268
Thermoleophilia	0.217	Leotiomycetes	-0.457*
AD3	-0.734**	Spizellomycetes	0.300
Nitrososphaeria	0.016	Mucoromycetes	0.258
Planctomycetes	0.326	Umbelopsidomycetes	-0.352
Acidimicrobiia	0.212	Rozellomycotina_cls_Incertae_sedis	0.335
Clostridia	0.629**	Saccharomycetes	0.286
JG30-KF-CM66	-0.693**	Geminibasidiomycetes	0.041
Bacteroidia	-0.610**	Pezizomycotina_cls_Incertae_sedis	0.252
Polyangia	-0.335	Microbotryomycetes	-0.438*

* Indicates significant correlation at *P* < 0.05; ** Indicates highly significant correlation at *P* < 0.01

## Discussion

### Effect of soil layer on soil organic carbon mineralization

SOC decomposition rates were significantly influenced by fluctuations in soil architecture, external carbon contributions, and microbial assemblage composition across various soil strata [23, 69, 79]. Multiple investigations have established that as soil depth increases, external C inputs decline substantially, while microbial community architecture and taxonomic composition undergo considerable changes. These depth-related fluctuations profoundly affect SOC storage and its breakdown processes [19, 64]. Our findings revealed that SOC decomposition rate decreased by 27%, 35%, 45% and 49% within the 20–40 cm, 40–60 cm, 60–80 cm and 80–100 cm horizons, respectively, compared to the surface horizon (0–20 cm; 842 mg kg-1; Fig. 1). This observation aligns with our initial hypothesis that SOC decomposition rate decline substantially with increasing soil depth, and these outcomes correspond with discoveries by Tian et al. [71], who established that during a 150-day laboratory incubation at both 20 °C and 25 °C, cumulative SOC decomposition remained consistently elevated in topsoil horizons, followed by midsoil and subsoil strata in a subtropical mixed forest ecosystems. Similarly, Wang et al. [78] documented cumulative CO_2_ emissions from northern peatland landscapes in Changbai Mountain, which exhibited consistent inverse relationships with increasing soil depth at incubation temperatures of 5 °C, 15 °C, and 25 °C.

Compared with traditional annual crops, tea plantations, as perennial cash crops, often use high-intensity pruning and high-dose organic fertilizer to improve yield and quality, thereby promoting SOC accumulation [82]. However, because deep plowing is insufficient, it is difficult to stably sequester large amounts of SOC, thereby limiting the C sink function of the tea plantation [74]. Our results indicated that the SOC decomposition rate decreased significantly with increasing depth, and that the C sequestration efficiency of deep soil was higher. Therefore, it is highly significant to implement deep plowing after pruning and fertilization to enhance SOC sequestration in tea plantations.

Mechanisms underlying the reduction in SOC decomposition rate with increasing soil depth may include the following three factors. (1) Organic amendments (e.g., organic fertilisers, pruned tea residues) applied to tea cultivation systems are primarily concentrated in the topsoil (0–20 cm). Although cultivation practices may promote some downward transport, SOC quantities in deeper soil strata remain substantially reduced. This vertical distribution of SOC availability directly constrains microbial substrate availability, thereby diminishing SOC decomposition rate in subsurface horizons [29, 55, 80]. This pattern becomes apparent in the substantial reductions in POC, MAOC, WSOC, and MBC concentrations with increasing soil depth (Fig. 2); (2) With progressive soil depth, declining SOC concentrations and available nutrients significantly affect microbial activity and composition, particularly the prevalence of functional microbes linked to C breakdown, leading to a reduction in SOC decomposition rate [49, 72]. This is corroborated by our findings that, in deeper soil strata within tea cultivation systems, microbial community architecture underwent considerable changes alongside a decline in the abundance of functional genes, including *GH48* and *cbh*I (Figs. 4 and 5, and 6); (3) Soil microbial community modifications with increasing depth also substantially reduce carbon-degrading enzyme activity, which contributes to the documented decline in SOC decomposition rate [41, 60].

### Relationships between the soil C pool and SOC mineralization

As the C source for SOC mineralization, SOC pools significantly influence the mineralization rate through their dynamic changes [40, 87]. Extensive literature has shown that the more labile organic C in soil, the higher its SOC mineralization rate, and that a higher percentage of recalcitrant SOC has a suppressive effect on SOC mineralization rate [3, 77]. We found that POC and MAOC contents in soil were significantly reduced with increasing soil depth in tea plantations (Figs. 2a-d and 7a-b), and both were positively related to SOC mineralization rate (Figs. 2a-d and 7a-b). As an unstable SOC pool, the content of POC is positively correlated with the mineralization rate of SOC [11, 39]. A higher percentage of POC considerably increases SOC mineralization, indicating its high bioavailability to micro-organisms [11, 39]. MAOC, formed through in vivo turnover and ex vivo chemical transformations, has a longer residence time and generally contributes less to mineralization, and a higher proportion of MAOC was associated with a lower SOC mineralization rate [14, 62]. These results indicate that the decrease in POC content with increasing soil depth is significantly associated with reduced SOC mineralization, whereas changes in MAOC content play a lesser role in controlling mineralization.

**Fig. 9:**
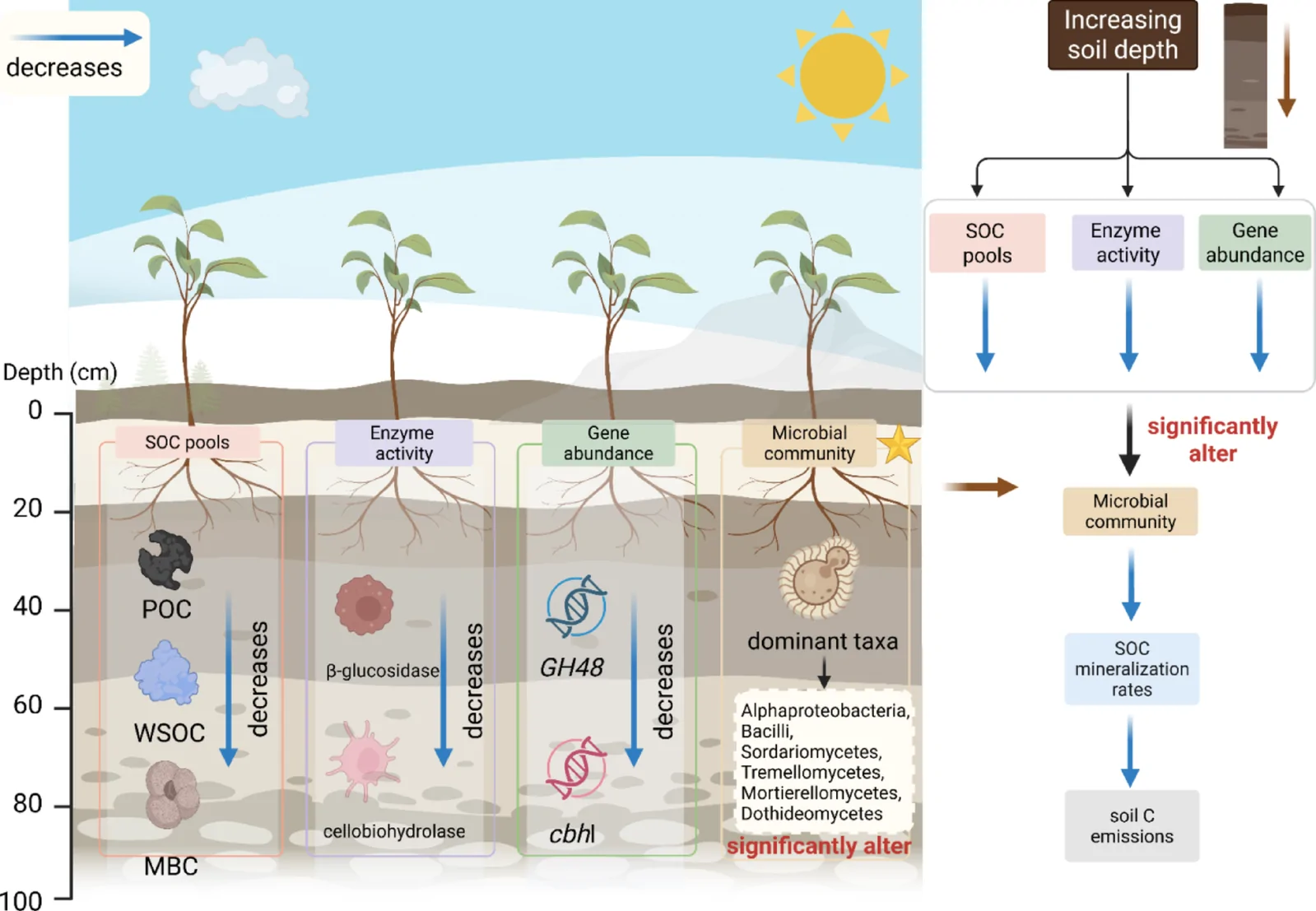
Concomitant shifts in microbial community composition and activity reduce soil organic carbon mineralization with increasing soil depth in tea plantations

In addition, the increase in WSOC and MBC content, as critical components of labile organic C in soils, provides sufficient C substrates for microbial metabolism, thereby accelerating the decomposition of SOC by microorganisms [18, 42]. Our results demonstrate that both WSOC and MBC decreased significantly with increasing soil depth and that these labile C fractions showed significant positive correlations with SOC mineralization rate (Figs. 2e-h and 7c-d). Therefore, we conclude that the observed decrease in SOC mineralization rate with increasing depth is significantly associated with decreases in WSOC and MBC content in the soil (Fig. 9).

### Relationships between soil microbial community and SOC mineralization

The soil microbial community is numerically vast and exhibits remarkably complex diversity, with structural and compositional changes directly governing SOC mineralization [28, 36, 54, 90, 93]. Our findings revealed that SOC decomposition showed no clear directional relationship with increasing soil depth for bacterial and fungal diversity (Shannon index) and richness (Chao1 index). This suggests that changes in microbial diversity and richness do not provide sufficient evidence to explain the observed variations in SOC decomposition.

Furthermore, our research establishes that variations among distinct SOC fractions drastically reshape microbial community structures and phylogenetic arrangements, especially the relative representation of dominant taxonomic groups (Figs. 5 and 6, and 8). These results correspond with previous investigations confirming that such taxonomic redistributions among critical microbial populations may profoundly affect both the breakdown and preservation of SOC [2, 70]. In particular, abundance modifications of major microbial functional clusters have been shown to have considerable impacts on SOC transformation processes [44, 75]. Remarkably, specific Alphaproteobacteria strains characterized as potential copiotrophs exhibit accelerated proliferation and are commonly described as “opportunistic” organisms, with increased abundances correlated with intensified SOC breakdown and expedited mineralization [15, 79]. In contrast, Bacilli actively decompose plant and animal detritus and therefore participate in fragmenting complex organic substances within soil matrices [51]. Our investigation revealed that the proportional abundances of both Alphaproteobacteria and Bacilli were substantially diminished with increasing soil depth (Fig. 6). Among these groups, particularly notable were strong positive associations of these bacterial assemblages with SOC mineralization rate, indicating their crucial roles in SOC breakdown processes across soil profiles (Table 2). These findings reinforce the concept that structural modifications in bacterial communities, particularly reductions in the abundance of predominant classes such as Alphaproteobacteria and Bacilli, substantially decrease SOC decomposition in deeper soil strata, thereby enhancing carbon sequestration potential.

Furthermore, our study demonstrates that fungal community composition undergoes depth-dependent changes, where proportional abundances of Sordariomycetes and Tremellomycetes decreased markedly with soil depth, while Mortierellomycetes and Dothideomycetes exhibited significantly ascending trends (Fig. 6). Simultaneously, SOC mineralization rate showed positive associations with Sordariomycetes and Tremellomycetes abundances, and negative relationships with Mortierellomycetes and Dothideomycetes abundances (Table 2). Research established that Sordariomycetes and Tremellomycetes fungi actively synthesise hydrolytic enzymes linked to SOC mineralization, and their elevated proportional abundances directly promote SOC mineralization rate [9, 94]. Additionally, our prior research established that Mortierellomycetes and Dothideomycetes demonstrate significant positive associations with SOC mineralization rate in tea plantation soils, with increased proportional abundances of these fungal assemblages being linked to SOC decomposition deceleration in tea plantation ecosystems [89]. These data indicate that SOC mineralization rate decrease with depth and are closely related to decreases in the abundance of the fungal classes Sordariomycetes and Tremellomycetes, and increases in the abundance of the classes Mortierellomycetes and Dothideomycetes. This underscores that microbial community restructuring (specifically functional transitions among key fungal taxa) is essential for regulating SOC decomposition in tea plantation soils (Fig. 9).

### The relationship between soil carbon-cycling enzyme activities, functional gene, and SOC mineralization

The presence of specific properties of a microbial community exerts a considerable influence on the activity of C-degrading enzymes and determines the rates of SOC mineralization [37, 66, 68, 83]. In our study, we discovered that both the activity of β-glucosidase and cellobiohydrolase in soil were significantly lower with increasing soil depth (Fig. 3a-d). Moreover, the rate of SOC mineralization also began to decline alongside the values of these two enzyme activities (Fig. 7e and f). Soil cellobiohydrolase is the key enzyme for degrading refractory crystalline cellulose, providing a substrate for β-glucosidase, and soil β-glucosidase is the final rate-limiting enzyme in soil polysaccharide degradation, converting cellobiose to available glucose. An increase in the activity of both enzymes will significantly promote the rate of SOC mineralisation [91]. With the increase of soil depth, the above two enzyme activities showed a significant decrease trend, which was accompanied by the decrease of SOC mineralization rate. The present finding is supported by similar findings by Xiao et al. [84] and Yang et al. [88], who also found that the SOC mineralization rate in the Mollisols and semi-arid grassland soils (under conservation tillage) significantly increased following an increase in the activity of β-glucosidase and cellobiohydrolase. These findings suggest that the pronounced decline in the mineralization rate of SOC with soil depth is significantly associated with a decrease in β-glucosidase and cellobiohydrolase in soil (Fig. 9).

Various research studies have shown that high copy numbers of functional genes involved in C degradation in the soil significantly enhance the mineralization of SOC [27, 95, 98]. Functional genes *GH48* and *cbh*I have a close connection with the SOC decomposition and have been generally concluded to enhance the SOC decomposition and stimulate soil C loss at high gene copy numbers [10, 16, 21, 76]. We observed that both *the GH48 and cbh*I genes decreased with soil depth in this study and showed a positive correlation with SOC mineralization rate (Figs. 4 and 7g-h). This observation indicates that a decline in the mineralization rate of SOC across the soil profile is in close association with reduced abundance of these C-degrading functional genes (*GH48* and *cbh*I) (Fig. 9).

## Conclusions

Our research established that SOC decomposition rate in tea-cultivation soils decrease systematically with increasing soil depth. The key driver of this decline is the combined influence of reduced labile C fractions (e.g., POC, WSOC and MBC), reduced C-degrading enzyme activities (e.g., β-glucosidase and cellobiohydrolase), and altered soil microbial community composition. Most notably, changes in the composition of soil microbial communities, particularly the relative abundance of microorganisms associated with C decomposition (e.g., Alphaproteobacteria and Bacilli), strongly influence SOC mineralization. these findings establish that changes in microbial community structure with increasing soil depth, especially the decline in the number of functional microbial taxa associated with C decomposition, effectively decelerate deep soil organic C decomposition. However, our findings focused solely on soil profiles and did not fully address spatial heterogeneity or the temporal dynamics of root secretion driven by specific tea garden management practices, such as fertilizer bands versus rows. To further elucidate the relevant processes, follow-up studies are recommended to explore organic carbon turnover across different soil layers, mediated by tea garden management measures, and to be validated in the field through isotopic tracing experiments and enzyme kinetics analyses.

## Supplementary Information

Below is the link to the electronic supplementary material.

Supplementary Material 1

## Data Availability

No datasets were generated or analyzed during the current study.
