# Supplementary material for: Microbial community changes mediate the decline in soil organic carbon mineralization with depth in tea plantations
Source: Carbon Balance Manag. 2026 Jul 13;21:128. doi: 10.1186/s13021-026-00485-7 (PMC13670659; doi:10.1186/s13021-026-00485-7)
Supplement: Supplementary file 1 [file 13021_2026_485_MOESM1_ESM.docx]

**Supplementary Information:**

**Microbial community changes mediate the decline in soil organic carbon mineralization with depth in tea plantations**

Shaobo Zhang^a,c,#^, Yifeng Xie^a,b,#^, Honbing Zhang^a^, Weizhen Wu^d^, Wenyan Han^a,c^, Gaoidi Zhu^b^, Qiuhong Wang^a,c^, Claudien Habimana Simbi^e^, Rongxiu Yin^f,*^, Xin Li^a,c,*^

^a^Tea Research Institute, Chinese Academy of Agricultural Sciences, Hangzhou 310000, China

^b^State Key Laboratory of Subtropical Silviculture, Zhejiang A & F University, Hangzhou 311300, China

^c^State Key Laboratory of Tea Plant Germplasm Innovation and Resource Utilization, Hangzhou 310000, China

^d^Qingyuan County Agriculture and Rural Bureau, LiShui 323000, China

^e^Agricultural Information Institute, Chinese Academy of Agricultural Sciences, Beijing 100081, China.

^f^Tea Research Institute, Guizhou Provincial Academy of Agricultural Sciences, Guiyang 550006. China

***** Corresponding author:

RXY: yrxzhr@126.com;

XL: lixin@ tricaas.com; Tel.: +86-571-866-50425; Fax.: +86-571-866-50425

Number of text pages: 1

Submitted to: *Carbon Balance and Management*

**Measurements of soil enzyme activity**

The soil β-glucosidase (EC 3.2.1.21) activity was determined by the method of Alef and Nannipieri (1995) after minor modifications. Briefly, 1 g of moist soil was placed into a centrifuge tube, followed by the addition of 4 mL of MUB (pH 6) and 1 mL of *p*-nitrophenyl-β-D-glucoside (25 mmol L^−1^), and the tube with the mixture was then incubated at 37 °C for 1 h. When the incubation procedure was finished, 1 mL of CaCl_2_ (0.5 mol L^–1^) and 4 mL of the Tris buffer (pH 12) was added into the tube. Then, the mixed solution was filtered through a 0.45-μm membrane filter (Millipore Corp., USA), and the color intensity of the filtrate was determined at 508 nm on a Hitachi 150-20 spectrophotometer (Hitachi Ltd., Tokyo, Japan). The β-glucosidase enzyme is expressed by the amount of *p*-nitrophenol produced per unit mass of soil per hour (nmol g^−1^ h^−1^).

The soil cellobiohydrolase (CBH; EC 3.2.1.91) activity was determined following the method of Zhang et al. (2017) after minor modifications. In brief, 1 g of fresh soil sample was weighed into a centrifuge tube, and then 4 mL of acetate buffer (pH 5.5, 10 mmol L^−1^) and 1 mL of 4-nitrophenyl-β-D-cellobioside solution was added. Then, the tube with the mixed solutions was incubated at 37 °C for 2 h. The reaction was terminated by adding 1 mL of CaCl_2_ solution (0.5 mol L^−1^) and 4 mL of NaOH solution (0.5 mol L^−1^). Then, the mixed solution was filtered through a 0.45-μm membrane filter (Millipore Corp., USA), and the color intensity of the filtrate was determined at 410 nm on a Hitachi 150-20 spectrophotometer (Hitachi Ltd., Tokyo, Japan). The cellobiohydrolase is expressed by the amount of 4-nitrophenyl-β-D-cellobioside consumed per unit mass of soil per hour (μmol g^−1^ h^−1^).

**Analyses of microbial C-cycling genes**

The Fast DNA SPIN Kit (MP Biomedicals, CA, USA) was used to extract soil DNA. Real-time PCR (RT-PCR) quantification was performed for *GH48* and *cbh*I. Primer pairs GH481F/GH481R (5′-RCATBTACGGBATGCACTGGCT)/(VCCGCC

CCCCABGMGTARTACC-3′) and fungcbhIF/fungcbhIR(5′-ACCAAYTGCTAYA

CIRGYAA)/(GCYTCCCAIATRTCCATC-3′) were used to amplify GH48 and *cbh*I, respectively. RT-PCR was performed using an ABI7300 fluorescent quantitative PCR instrument (Applied Biosystems, USA). The SYBR green method was adopted for determining the abundance of three microbial functional genes using the following parameters: *GH48*: 95°C initial denaturation for 2 min, followed by 46 cycles at 95°C (30 s), 50°C (30 s), and 72°C (10 min + plate read); *cbh*I: 95°C initial denaturation for 5 min, followed by 40 cycles at 94°C (30 s), 48°C (30 s), and 72°C (90 s + plate read). Standard curves were generated using purified template plasmid DNA, and the abundance of the two aforementioned microbial functional genes in each soil sample (normalized to dry soil mass) was calculated.

**References**

Alef K, Nannipieri P. 1995. 7 - Enzyme activities, In: Alef, K., Nannipieri, P. (Eds.), Methods in Applied Soil Microbiology and Biochemistry. Academic Press, London, pp: 311-373.

Casida L E J, Klein D A, Santoro T. 1964. Soil dehydrogenase activity. Soil Science. 98: 371-376.

Zhang W W, Lu Z T, Yang K, Zhu J J. 2017. Impacts of conversion from secondary forests to larch plantations on the structure and function of microbial communities. Applied Soil Ecology. 111: 73-83.
